# 2,4,5-Tris(pyridin-4-yl)-1*H*-imidazole monohydrate

**DOI:** 10.1107/S1600536811053013

**Published:** 2011-12-14

**Authors:** Shen-Tang Wang, Guang-Bo Che, Chun-Bo Liu, Xing Wang, Ling Liu

**Affiliations:** a School of Chemistry and Chemical Engineering, Jiangsu University, Zhenjiang 212013, People’s Republic of China

## Abstract

In the crystal structure of the title compound, C_18_H_13_N_5_·H_2_O, adjacent mol­ecules are linked by O—H⋯N and N—H⋯O hydrogen bonds, generating a chain propagating along [001].

## Related literature

For the use of 2,4,5-tri(4-pyrid­yl)imidazole in the construction of metal-organic coordination polymers, see: Wang *et al.* (2009 ▶); Liang *et al.* (2009 ▶). For related structures, see: Jiang & Hou (2011 ▶); Li (2011 ▶); Li & Xia (2011 ▶). For the preparation, see: Proskurnina *et al.* (2002 ▶).
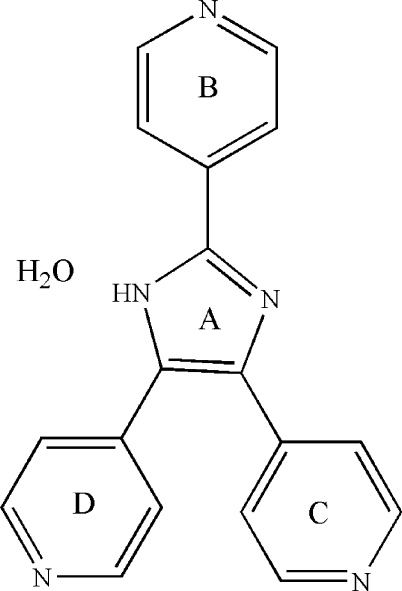


## Experimental

### 

#### Crystal data

C_18_H_13_N_5_·H_2_O
*M* *_r_* = 317.35
Triclinic, 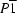

*a* = 8.1510 (16) Å
*b* = 9.5210 (19) Å
*c* = 11.506 (2) Å
α = 103.80 (3)°
β = 105.64 (3)°
γ = 101.03 (3)°
*V* = 803.3 (4) Å^3^
*Z* = 2
Mo *K*α radiation
μ = 0.09 mm^−1^
*T* = 293 K
0.35 × 0.25 × 0.2 mm

#### Data collection

Bruker SMART diffractometer
Absorption correction: multi-scan (*SADABS*; Bruker, 2002 ▶) *T*_min_ = 0.970, *T*_max_ = 1.000
7510 measured reflections
2912 independent reflections
1792 reflections with *I* > 2σ(*I*)
*R*_int_ = 0.040

#### Refinement

*R*[*F* ^2^ > 2σ(*F* ^2^)] = 0.058
*wR*(*F* ^2^) = 0.125
*S* = 1.02
2876 reflections
218 parameters
H-atom parameters constrained
Δρ_max_ = 0.19 e Å^−3^
Δρ_min_ = −0.17 e Å^−3^

### 

Data collection: *SMART* (Bruker, 2002 ▶); cell refinement: *SAINT* (Bruker, 2002 ▶); data reduction: *SAINT*; program(s) used to solve structure: *SHELXS97* (Sheldrick, 2008 ▶); program(s) used to refine structure: *SHELXL97* (Sheldrick, 2008 ▶); molecular graphics: *SHELXTL* (Sheldrick, 2008 ▶) and *DIAMOND* (Brandenburg, 1998 ▶); software used to prepare material for publication: *SHELXTL*.

## Supplementary Material

Crystal structure: contains datablock(s) global, I. DOI: 10.1107/S1600536811053013/zj2036sup1.cif

Structure factors: contains datablock(s) I. DOI: 10.1107/S1600536811053013/zj2036Isup2.hkl

Supplementary material file. DOI: 10.1107/S1600536811053013/zj2036Isup3.cml

Additional supplementary materials:  crystallographic information; 3D view; checkCIF report

## Figures and Tables

**Table 1:** Hydrogen-bond geometry (Å, °)

*D*—H⋯*A*	*D*—H	H⋯*A*	*D*⋯*A*	*D*—H⋯*A*
O1—H1*A*⋯N5^i^	0.85	1.99	2.843 (3)	177
N1—H1⋯O1	0.89	1.85	2.741 (3)	176
O1—H1*B*⋯N4^ii^	0.85	1.94	2.787 (3)	172

Symmetry codes: (i) 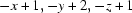; (ii) 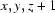.

**Fig. 1.**
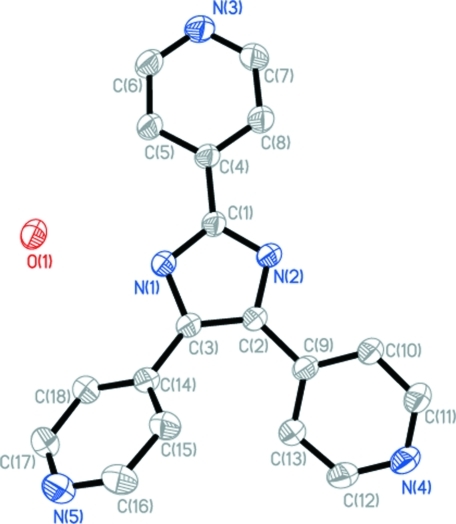
The molecular structure of the title compound with the atom numbering scheme. Displacement ellipsoids are drawn at the 30% probability level. All H atoms are presented as a small spheres of arbitrary radius.

**Fig. 2.**
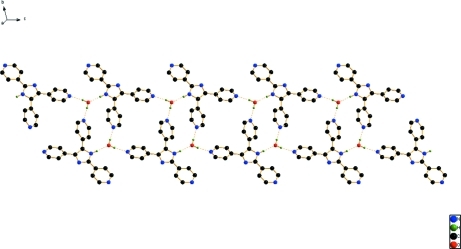
The one-dimensional superamolecular structure linked by the hydrogen bonds.

## supplementary crystallographic information

### Comment

In the 2,4,5-tri(4-pyridyl)imidazole three pyridyl groups (pyridyl ring B (C4-C8 and N3), C (C9-C13 and N4), D (C14-C18 and N5)) are directly connected with the imidazole ring A (C1-C3 , N1 and N2). The dihedral angles between the mean planes of pyridyl ring B and imidazole ring A, pyridyl ring C and imidazole ring A, and pyridyl ring D and imidazole ring A are 9.1 (7) °, 21.5 (5) °, 45.5 (1) °, respectively.

We report herein on the crystal structure of the title compound (Fig. 1). In the crystal lattice the molecules are linked by O—H···N and N—H···O hydrogen bonds (Jiang *et al.* 2011; Li *et al.* 2011; Li 2011) interactions to generate a one-dimensional double chain structure (Fig. 2).

### Experimental

The 2,4,5-tri(4-pyridyl)imidazole was prepared by the methord reported in the literature (Proskurnina *et al.* 2002). A mixture of 2,4,5-tri(4-pyridyl)imidazole (0.030 g, 0.1 mmol), 2 drops of 1 mol/L HCl and water (10 mL) was placed in a 25 mL Teflon-lined autoclave and heated for 3 d at 433 K under autogenous pressure. Upon cooling and opening the bomb, colourless block-shaped crystals were obtained, then washed with water and dried in air.

### Refinement

All H atoms on C atoms were positioned geometrically and refined as riding atoms, with (C—H = 0.93 Å) and refined as riding, with U_iso_(H)= 1.2 U_eq_(C). The hydrogen atoms of water molecules were located in a difference Fourier map, and were refined with suitable O—H distance restraint; U_iso_ = 1.5 U_eq_(O).

### Crystal data

**Table:**

C_18_H_13_N_5_·H_2_O	*Z* = 2
*M**_r_* = 317.35	*F*(000) = 332
Triclinic, *P*1	*D*_x_ = 1.312 Mg m^−^^3^
Hall symbol: -P 1	Mo *K*α radiation, λ = 0.71073 Å
*a* = 8.1510 (16) Å	Cell parameters from 3107 reflections
*b* = 9.5210 (19) Å	θ = 3.0–25.2°
*c* = 11.506 (2) Å	µ = 0.09 mm^−^^1^
α = 103.80 (3)°	*T* = 293 K
β = 105.64 (3)°	Prism, colourless
γ = 101.03 (3)°	0.35 × 0.25 × 0.2 mm
*V* = 803.3 (4) Å^3^	

### Data collection

**Table:**

Bruker SMART diffractometer	2912 independent reflections
Radiation source: fine-focus sealed tube	1792 reflections with *I* > 2σ(*I*)
graphite	*R*_int_ = 0.040
ω scans	θ_max_ = 25.2°, θ_min_ = 3.0°
Absorption correction: multi-scan (*SADABS*; Bruker, 2002)	*h* = −9→9
*T*_min_ = 0.970, *T*_max_ = 1.000	*k* = −11→11
7510 measured reflections	*l* = −13→13

### Refinement

**Table:**

Refinement on *F*^2^	Primary atom site location: structure-invariant direct methods
Least-squares matrix: full	Secondary atom site location: difference Fourier map
*R*[*F*^2^ > 2σ(*F*^2^)] = 0.058	Hydrogen site location: inferred from neighbouring sites
*wR*(*F*^2^) = 0.125	H-atom parameters constrained
*S* = 1.02	*w* = 1/[σ^2^(*F*_o_^2^) + (0.0171*P*)^2^ + 0.605*P*] where *P* = (*F*_o_^2^ + 2*F*_c_^2^)/3
2876 reflections	(Δ/σ)_max_ < 0.001
218 parameters	Δρ_max_ = 0.19 e Å^−^^3^
0 restraints	Δρ_min_ = −0.17 e Å^−^^3^

### Special details

**Table:**

Geometry. All esds (except the esd in the dihedral angle between two l.s. planes) are estimated using the full covariance matrix. The cell esds are taken into account individually in the estimation of esds in distances, angles and torsion angles; correlations between esds in cell parameters are only used when they are defined by crystal symmetry. An approximate (isotropic) treatment of cell esds is used for estimating esds involving l.s. planes.
Refinement. Refinement of F^2^ against ALL reflections. The weighted R-factor wR and goodness of fit S are based on F^2^, conventional R-factors R are based on F, with F set to zero for negative F^2^. The threshold expression of F^2^ > 2sigma(F^2^) is used only for calculating R-factors(gt) etc. and is not relevant to the choice of reflections for refinement. R-factors based on F^2^ are statistically about twice as large as those based on F, and R- factors based on ALL data will be even larger.

### Fractional atomic coordinates and isotropic or equivalent isotropic displacement parameters (Å^2^)

**Table:**

	*x*	*y*	*z*	*U*_iso_*/*U*_eq_	
N1	0.2548 (3)	0.5006 (2)	0.26415 (19)	0.0544 (6)	
H1	0.2800	0.5437	0.3469	0.082*	
N2	0.1414 (3)	0.3416 (2)	0.06806 (19)	0.0542 (6)	
N3	−0.0531 (4)	0.0012 (3)	0.3185 (2)	0.0738 (7)	
N4	0.2559 (4)	0.5283 (3)	−0.2901 (2)	0.0749 (7)	
N5	0.5288 (4)	1.0560 (3)	0.3379 (3)	0.0764 (8)	
C1	0.1603 (3)	0.3582 (3)	0.1895 (2)	0.0518 (6)	
C2	0.2265 (3)	0.4798 (3)	0.0649 (2)	0.0523 (6)	
C3	0.2960 (3)	0.5811 (3)	0.1858 (2)	0.0530 (7)	
C4	0.0870 (3)	0.2404 (3)	0.2365 (2)	0.0520 (6)	
C5	0.1271 (4)	0.2521 (3)	0.3637 (3)	0.0671 (8)	
H5A	0.2026	0.3399	0.4252	0.080*	
C6	0.0536 (4)	0.1317 (3)	0.3987 (3)	0.0739 (9)	
H6A	0.0812	0.1433	0.4849	0.089*	
C7	−0.0932 (4)	−0.0074 (3)	0.1966 (3)	0.0754 (9)	
H7A	−0.1703	−0.0962	0.1374	0.091*	
C8	−0.0284 (4)	0.1062 (3)	0.1517 (3)	0.0668 (8)	
H8A	−0.0621	0.0926	0.0650	0.080*	
C9	0.2386 (3)	0.4986 (3)	−0.0558 (2)	0.0527 (6)	
C10	0.1214 (4)	0.4006 (3)	−0.1700 (2)	0.0594 (7)	
H10A	0.0342	0.3211	−0.1709	0.071*	
C11	0.1334 (4)	0.4203 (3)	−0.2821 (3)	0.0708 (8)	
H11A	0.0505	0.3537	−0.3572	0.085*	
C12	0.3716 (4)	0.6201 (4)	−0.1801 (3)	0.0775 (9)	
H12A	0.4600	0.6962	−0.1826	0.093*	
C13	0.3698 (4)	0.6105 (3)	−0.0626 (3)	0.0681 (8)	
H13A	0.4552	0.6778	0.0110	0.082*	
C14	0.3805 (4)	0.7431 (3)	0.2368 (2)	0.0549 (7)	
C15	0.3141 (4)	0.8416 (3)	0.1767 (3)	0.0675 (8)	
H15A	0.2185	0.8049	0.1015	0.081*	
C16	0.3925 (4)	0.9939 (3)	0.2305 (3)	0.0754 (9)	
H16A	0.3470	1.0577	0.1890	0.090*	
C17	0.5915 (4)	0.9608 (3)	0.3939 (3)	0.0735 (9)	
H17A	0.6879	1.0009	0.4685	0.088*	
C18	0.5219 (4)	0.8055 (3)	0.3480 (3)	0.0650 (8)	
H18A	0.5701	0.7446	0.3918	0.078*	
O1	0.3155 (4)	0.6367 (2)	0.51602 (18)	0.1058 (10)	
H1A	0.3597	0.7298	0.5581	0.159*	
H1B	0.3044	0.5992	0.5745	0.159*	

### Atomic displacement parameters (Å^2^)

**Table:**

	*U*^11^	*U*^22^	*U*^33^	*U*^12^	*U*^13^	*U*^23^
N1	0.0703 (14)	0.0475 (12)	0.0430 (12)	0.0090 (10)	0.0198 (10)	0.0139 (9)
N2	0.0711 (15)	0.0475 (12)	0.0439 (12)	0.0088 (10)	0.0215 (11)	0.0167 (9)
N3	0.0983 (19)	0.0555 (14)	0.0683 (17)	0.0082 (13)	0.0323 (15)	0.0249 (13)
N4	0.102 (2)	0.0736 (16)	0.0558 (15)	0.0144 (15)	0.0375 (15)	0.0265 (13)
N5	0.0884 (19)	0.0549 (15)	0.0791 (18)	0.0055 (14)	0.0317 (16)	0.0152 (14)
C1	0.0689 (17)	0.0422 (13)	0.0430 (14)	0.0118 (12)	0.0187 (13)	0.0129 (11)
C2	0.0652 (16)	0.0482 (14)	0.0426 (14)	0.0091 (12)	0.0199 (12)	0.0153 (11)
C3	0.0660 (17)	0.0482 (14)	0.0446 (14)	0.0091 (12)	0.0201 (13)	0.0171 (11)
C4	0.0680 (17)	0.0428 (13)	0.0470 (15)	0.0122 (12)	0.0209 (13)	0.0174 (11)
C5	0.097 (2)	0.0510 (15)	0.0484 (16)	0.0064 (15)	0.0245 (15)	0.0162 (13)
C6	0.111 (3)	0.0599 (18)	0.0564 (18)	0.0163 (17)	0.0360 (18)	0.0236 (15)
C7	0.094 (2)	0.0519 (17)	0.068 (2)	−0.0007 (15)	0.0184 (17)	0.0212 (15)
C8	0.088 (2)	0.0522 (16)	0.0527 (16)	0.0074 (15)	0.0191 (15)	0.0174 (13)
C9	0.0659 (17)	0.0511 (14)	0.0462 (14)	0.0145 (12)	0.0225 (13)	0.0204 (12)
C10	0.0738 (18)	0.0566 (16)	0.0473 (15)	0.0113 (14)	0.0228 (14)	0.0169 (13)
C11	0.090 (2)	0.0732 (19)	0.0474 (17)	0.0160 (17)	0.0259 (16)	0.0173 (14)
C12	0.097 (2)	0.071 (2)	0.068 (2)	0.0054 (17)	0.0402 (19)	0.0273 (17)
C13	0.079 (2)	0.0656 (18)	0.0517 (17)	0.0000 (15)	0.0251 (15)	0.0158 (14)
C14	0.0670 (17)	0.0486 (14)	0.0492 (15)	0.0082 (12)	0.0251 (13)	0.0148 (12)
C15	0.078 (2)	0.0519 (16)	0.0657 (19)	0.0083 (14)	0.0172 (16)	0.0212 (14)
C16	0.087 (2)	0.0536 (17)	0.086 (2)	0.0151 (16)	0.0285 (19)	0.0265 (16)
C17	0.080 (2)	0.0604 (18)	0.0624 (19)	−0.0019 (16)	0.0216 (16)	0.0061 (15)
C18	0.077 (2)	0.0548 (16)	0.0552 (17)	0.0068 (14)	0.0185 (15)	0.0156 (13)
O1	0.187 (3)	0.0597 (13)	0.0500 (12)	−0.0125 (14)	0.0451 (14)	0.0087 (10)

### Geometric parameters (Å, °)

**Table:**

N1—C1	1.363 (3)	C7—H7A	0.9300
N1—C3	1.381 (3)	C8—H8A	0.9300
N1—H1	0.8907	C9—C10	1.381 (3)
N2—C1	1.331 (3)	C9—C13	1.390 (3)
N2—C2	1.379 (3)	C10—C11	1.373 (4)
N3—C6	1.326 (4)	C10—H10A	0.9300
N3—C7	1.329 (4)	C11—H11A	0.9300
N4—C11	1.329 (4)	C12—C13	1.381 (4)
N4—C12	1.330 (4)	C12—H12A	0.9300
N5—C16	1.330 (4)	C13—H13A	0.9300
N5—C17	1.333 (4)	C14—C18	1.378 (4)
C1—C4	1.454 (3)	C14—C15	1.397 (4)
C2—C3	1.383 (3)	C15—C16	1.378 (4)
C2—C9	1.469 (3)	C15—H15A	0.9300
C3—C14	1.463 (3)	C16—H16A	0.9300
C4—C5	1.383 (3)	C17—C18	1.390 (4)
C4—C8	1.385 (3)	C17—H17A	0.9300
C5—C6	1.387 (4)	C18—H18A	0.9300
C5—H5A	0.9300	O1—H1A	0.8543
C6—H6A	0.9300	O1—H1B	0.8500
C7—C8	1.382 (4)		
			
C1—N1—C3	107.5 (2)	C10—C9—C13	116.4 (2)
C1—N1—H1	130.1	C10—C9—C2	120.8 (2)
C3—N1—H1	122.1	C13—C9—C2	122.8 (2)
C1—N2—C2	105.6 (2)	C11—C10—C9	120.2 (3)
C6—N3—C7	115.0 (2)	C11—C10—H10A	119.9
C11—N4—C12	115.6 (2)	C9—C10—H10A	119.9
C16—N5—C17	116.0 (3)	N4—C11—C10	124.1 (3)
N2—C1—N1	111.3 (2)	N4—C11—H11A	117.9
N2—C1—C4	124.4 (2)	C10—C11—H11A	117.9
N1—C1—C4	124.3 (2)	N4—C12—C13	124.6 (3)
N2—C2—C3	110.2 (2)	N4—C12—H12A	117.7
N2—C2—C9	119.8 (2)	C13—C12—H12A	117.7
C3—C2—C9	130.0 (2)	C12—C13—C9	119.1 (3)
N1—C3—C2	105.3 (2)	C12—C13—H13A	120.5
N1—C3—C14	120.4 (2)	C9—C13—H13A	120.5
C2—C3—C14	134.0 (2)	C18—C14—C15	117.3 (2)
C5—C4—C8	116.3 (2)	C18—C14—C3	122.0 (2)
C5—C4—C1	123.8 (2)	C15—C14—C3	120.6 (2)
C8—C4—C1	119.9 (2)	C16—C15—C14	118.9 (3)
C4—C5—C6	119.4 (3)	C16—C15—H15A	120.5
C4—C5—H5A	120.3	C14—C15—H15A	120.5
C6—C5—H5A	120.3	N5—C16—C15	124.5 (3)
N3—C6—C5	124.9 (3)	N5—C16—H16A	117.7
N3—C6—H6A	117.5	C15—C16—H16A	117.7
C5—C6—H6A	117.5	N5—C17—C18	124.1 (3)
N3—C7—C8	124.6 (3)	N5—C17—H17A	117.9
N3—C7—H7A	117.7	C18—C17—H17A	117.9
C8—C7—H7A	117.7	C14—C18—C17	119.1 (3)
C7—C8—C4	119.7 (3)	C14—C18—H18A	120.4
C7—C8—H8A	120.1	C17—C18—H18A	120.4
C4—C8—H8A	120.1	H1A—O1—H1B	100.9
			
C2—N2—C1—N1	−0.5 (3)	N2—C2—C9—C10	21.5 (4)
C2—N2—C1—C4	178.6 (3)	C3—C2—C9—C10	−161.6 (3)
C3—N1—C1—N2	1.3 (3)	N2—C2—C9—C13	−156.1 (3)
C3—N1—C1—C4	−177.8 (3)	C3—C2—C9—C13	20.8 (5)
C1—N2—C2—C3	−0.5 (3)	C13—C9—C10—C11	−2.7 (4)
C1—N2—C2—C9	176.9 (2)	C2—C9—C10—C11	179.5 (3)
C1—N1—C3—C2	−1.6 (3)	C12—N4—C11—C10	0.2 (5)
C1—N1—C3—C14	173.1 (2)	C9—C10—C11—N4	1.6 (5)
N2—C2—C3—N1	1.3 (3)	C11—N4—C12—C13	−0.8 (5)
C9—C2—C3—N1	−175.8 (3)	N4—C12—C13—C9	−0.5 (5)
N2—C2—C3—C14	−172.3 (3)	C10—C9—C13—C12	2.2 (4)
C9—C2—C3—C14	10.6 (5)	C2—C9—C13—C12	179.9 (3)
N2—C1—C4—C5	170.9 (3)	N1—C3—C14—C18	45.8 (4)
N1—C1—C4—C5	−10.2 (4)	C2—C3—C14—C18	−141.4 (3)
N2—C1—C4—C8	−8.3 (4)	N1—C3—C14—C15	−131.1 (3)
N1—C1—C4—C8	170.7 (3)	C2—C3—C14—C15	41.7 (5)
C8—C4—C5—C6	0.8 (4)	C18—C14—C15—C16	0.2 (4)
C1—C4—C5—C6	−178.4 (3)	C3—C14—C15—C16	177.3 (3)
C7—N3—C6—C5	−2.3 (5)	C17—N5—C16—C15	0.7 (5)
C4—C5—C6—N3	1.1 (5)	C14—C15—C16—N5	−0.3 (5)
C6—N3—C7—C8	1.7 (5)	C16—N5—C17—C18	−0.9 (5)
N3—C7—C8—C4	0.1 (5)	C15—C14—C18—C17	−0.4 (4)
C5—C4—C8—C7	−1.3 (4)	C3—C14—C18—C17	−177.4 (3)
C1—C4—C8—C7	177.9 (3)	N5—C17—C18—C14	0.8 (5)

### Hydrogen-bond geometry (Å, °)

**Table:**

*D*—H···*A*	*D*—H	H···*A*	*D*···*A*	*D*—H···*A*
O1—H1A···N5^i^	0.85	1.99	2.843 (3)	177.
N1—H1···O1	0.89	1.85	2.741 (3)	176.
O1—H1B···N4^ii^	0.85	1.94	2.787 (3)	172.

Symmetry codes: (i) −*x*+1, −*y*+2, −*z*+1; (ii) *x*, *y*, *z*+1.
